# Bronchial thermoplasty increases airway volume measured by functional respiratory imaging

**DOI:** 10.1186/s12931-019-1132-9

**Published:** 2019-07-16

**Authors:** D. Langton, G. Sloan, C. Banks, K. Bennetts, V. Plummer, F. Thien

**Affiliations:** 1 Department of Thoracic Medicine, Frankston Hospital, Peninsula Health, 2 Hastings Road, Frankston, VIC 3199 Australia; 2 Faculty of Medicine, Nursing and Health Sciences, Monash University, Clayton, Vic Australia; 3 Department of Medical Imaging, Frankston Hospital, Peninsula Health, Frankston, Vic Australia; 4 Department of Respiratory Medicine, Eastern Health, Box Hill, Vic Australia

**Keywords:** Asthma, Bronchial thermoplasty, Airway volume

## Abstract

**Background:**

The purpose of this study was to use CT scanning with computational fluid dynamics to evaluate the mechanisms by which Bronchial Thermoplasty (BT) improves asthmatic symptoms.

**Methods:**

The study was conducted in a university teaching hospital, experienced in performing BT. Imaging studies were performed before, and after, BT of the left lung, and prior to treatment of the right lung, which therefore acted as a control. On each occasion, two high-resolution CT scans were performed, one at full inspiration (TLC) and the other at Functional Residual Capacity (FRC). The study protocol was offered to 10 patients, all of whom met the definition of severe asthma, despite high dose inhaled corticosteroids and dual long acting bronchodilators.

**Results:**

Significant increases in airway luminal volume were observed on the treated side, compared with control, at both full inspiration (by 27%) and at FRC (by 17%). The ratio of distal airway volume to lung volume significantly increased on the treated side. The change in airway volume with inspiration from FRC increased by 48% on the treated side compared to 5% in the control lung, suggesting treatment increased airway distensibility. No effect was observed on airway wall thickness, nor air trapping. There was a trend towards correlation between the improvement in airway volume at TLC and improvement in symptoms.

**Conclusion:**

This study demonstrates that BT increases the luminal airway volume on the treated side compared to the control lung. We suggest that this is an important link between the airway smooth muscle atrophy demonstrated pathologically, and the improvement in symptoms observed clinically.

## Summary at a glance

Functional Respiratory Imaging using CT scanning was used to evaluate the effect of bronchial thermoplasty on one lung, whilst the other served as a control. On the treated side, airway luminal volume substantially increased.

## Background

Whilst most patients with asthma can expect to be well controlled with inhaled corticosteroids and bronchodilators [1], there remains a small group of patients, estimated to account for 5–10% of adults with asthma, with more refractory, severe disease [2]. These patients have daily symptoms that limit their quality of life, and they have frequent exacerbations which result in lost productivity and increased healthcare utilisation [3]. There are limited therapeutic options for such patients who often find themselves treated with chronic oral steroids with all the attendant steroid related side effects [4]. Hence, there is a need to explore potential alternative treatment options.

Bronchial thermoplasty (BT) is one such option. It was designed to address the known fact that in asthma the airway smooth muscle is hypertrophied [5], and this causes bronchoconstriction and hence wheezing. BT uses a radiofrequency catheter to cause atrophy of this airway smooth muscle [6]. This has been proven to reduce asthma symptoms and asthma exacerbations in randomised control trials [7–9], but the improvements in lung function which might have been anticipated with such treatment, have either been non-existent [7–9] or modest [10]. Therefore, there is a need to better understand the mechanisms by which this treatment works.

An emerging new field in the evaluation of lung disease is functional imaging, as opposed to purely anatomical imaging [11]. Three techniques exist, namely (i) computerised tomography (CT) (ii) single photon emission computerised tomography (SPECT) and (iii) Magnetic Resonance Imaging (MRI) with a hyperpolarised gas such as helium-3 and xenon-129. Whilst SPECT and MRI provide a detailed evaluation of functional ventilation heterogeneity, they lack the anatomical precision of CT scanning in relation to identification of airway dimensions. A further advantage of using CT scanning is that images can be acquired both at full inspiration and at end expiration, and then computational fluid dynamics can be used to model the dynamic changes in the lung during respiration [12–16].

The purpose of this study was to use CT scanning with computational fluid dynamics to learn more about the mechanisms by which BT improves asthma symptoms, particularly insofar as altering airway and lobar dimensions.

## Method

### Setting and study design

This study was conducted in a university teaching hospital with a dedicated severe asthma clinic and five years’ experience in performing BT. The schedule of the BT procedures was altered in a novel way in order to achieve one treated lung (left side) and one untreated lung (right side). The left lower lobe was treated in the first BT session, and then the left upper lobe in the second session. Imaging studies were conducted at baseline, and then again four weeks after completion of the left upper lobe BT, and prior to treatment of the right lung (which acted as a control). Following the second set of imaging, the right lower and upper lobes were treated together in the final BT session.

### Participants

The study protocol was offered to 10 patients with severe asthma who had already chosen to undergo BT. The participants had been thoroughly evaluated and found to meet the ERS/ATS definition of severe asthma [17], despite using substantive asthma therapy including high dose inhaled corticosteroids and dual long acting bronchodilators. Alternative respiratory conditions such as COPD or bronchiectasis had been excluded. All 10 patients had previously been evaluated for monoclonal antibody therapy. Six did not meet Australian funding guidelines (e.g. low eosinophil count), three had previously been treated with omalizumab or mepolizumab, which then had been ceased because of lack of effect, and one patient continued to have severe poorly controlled asthma despite 12 months continuing therapy with mepolizumab. Women of reproductive age who were not using highly effective methods of contraception were excluded from participation so as to avoid radiation exposure to an unborn child.

### Imaging studies

Non-contrast CT Scanning was performed on a 128-slice Siemens Definition AS+ scanner with a helical slice thickness of 0.6 mm, rotation time of 0.6 s, detector coverage of 38.4 mm, and tube voltage of 100 kV, consistent with the previously published technique for Functional Respiratory Imaging [13]. A typical scan was completed in under 5 s. Two breath-hold scans were performed on each occasion – one at full inspiration, and the other at Functional Residual Capacity (FRC). Immediately prior to the CT scan, the patient was coached in the manoeuvres required for the breath-hold, and a member of the research team was present during the scan, observing the patient from the control room and providing instruction. All imaging was performed in a stable state, pre-bronchodilator, and prior to peri-procedural oral steroid administration. The average estimated radiation exposure for each CT scanning session (comprising 2 scans) was 4.6 mSv, or 9.2 mSv radiation exposure for the whole study. A third CT analysis was envisaged after all BT treatment, but at the request of the Ethics Committee this was deferred by 12 months in order to remain within safe annual radiation exposure limits. When available, this data will be presented in a future manuscript.

Post acquisition, CT images were analysed independently to the investigating team by FLUIDDA (Kontich, Belgium). The high-resolution images were imported into Mimics, a commercial, medical imaging processing software package (Materialise, Leuvin, Belgium), which converted the CT images into patient-specific, 3D computer models of the lung lobes and the airway dimensions. Subsequent mathematical modelling was then performed by FLUIDDA, who reported the following parameters for each lobe of each lung: (i) lobar volume (ii) airway volume (iii) airway resistance (iv) airway wall thickness (v) and air trapping.

### Clinical measurements

The baseline data routinely recorded for all BT patients included age, gender, weight, height, asthma medication usage, asthma exacerbation history, lung function parameters and the asthma quality of life score, the ACQ-5 [18]. Written permission to use the ACQ-5 was provided by the author, Elizabeth Juniper. Patient assessments were performed by experienced clinical research nursing and scientific staff, and were conducted independently of the procedural team. Spirometry and body plethysmography were performed using the Jaegar Masterscreen Body (Carefusion, Hoechberg, Germany) and tests were conducted in the morning, and having withheld bronchodilators since the previous evening. The laboratory equipment was calibrated on the morning of testing and all tests were conducted to ERS/ATS standards [19]. The predicted value equations used were taken from the Global Lung Initiative [20]. Patient assessments were conducted at baseline, mid–treatment with the left lung treated and right lung untreated, and then at 6 weeks after all treatments were completed.

### Safety

All patients undergoing BT at our institution are routinely observed overnight following their procedure. An adverse event is recorded wherever a patient remains in hospital longer than the pre-planned 24 h, or is readmitted to hospital for any reason within 30 days of a BT procedure.

### Statistical analysis

SPSS version 25 (IBM corporation, New York, USA) was used for all statistical analyses. Grouped data is reported as mean ± standard deviation, excepting where the data is not normally distributed, in which case median and interquartile range are used. A paired t- test was used to compare the results for post BT with pre BT, or if there were multiple sets of repeated measured, analysis of variance (ANOVA) was used. Statistical significance was taken at *p* < 0.05 for a two-tailed test.

### Ethics

Prospective approval to undertake this study was provided by the Peninsula Health Human Research Ethics Committee and no patient was enrolled without having given informed consent.

## Results

### Baseline characteristics

Seven females and three males participated, mean age 62.2 ± 7.7 years, body mass index 30.6 ± 6.2 kg/m^2^. The mean Forced Expiratory Volume in one second (FEV1) was 42.9 ± 11.5% predicted, with an improvement in FEV1 following 400μg of salbutamol of 9.6 ± 9.2%. The mean forced expiratory ratio was 47.4 ± 10.7%. The mean Total Lung Capacity (TLC) was 108.3 ± 20.7% predicted, and Residual Volume (RV) was 159 ± 49% predicted. The average diffusion capacity for carbon monoxide was 83.3 ± 32.7% predicted.

The average ACQ-5 score was 3.4 ± 1.0. The median daily requirement for short acting beta-2 agonists was 6.75 puffs (1,20). Eighty per cent of patients were taking maintenance oral corticosteroids, mean dose 6.7 ± 7.3 mg/day. All patients were using dual long acting bronchodilators, as well as inhaled corticosteroids, mean dose 1500 ± 850 μg/day in beclomethasone equivalence. In the 6 months prior to BT, the average number of exacerbations requiring an increase in oral corticosteroids by more than 10 mg/day was 2.2 ± 1.4. The mean serum eosinophil count was 200 ± 100 cells/ul, and the median IgE was 13 (3,187) IU/ml. Five patients had never smoked and five were former smokers.

### Treatment

The average number of radiofrequency activations administered to the left lung was 117 ± 21, and to the right lung was 82 ± 23. In the thirty procedures, on two occasions patients remained in hospital longer than expected, and both occurred after the consolidated treatment of the right lung. In both cases the issue was wheezing and one of these patients required a short period of non-invasive ventilation. There were no readmissions to hospital for any cause within 30 days of a BT procedure.

### Response to treatment

**Fig. 1:**
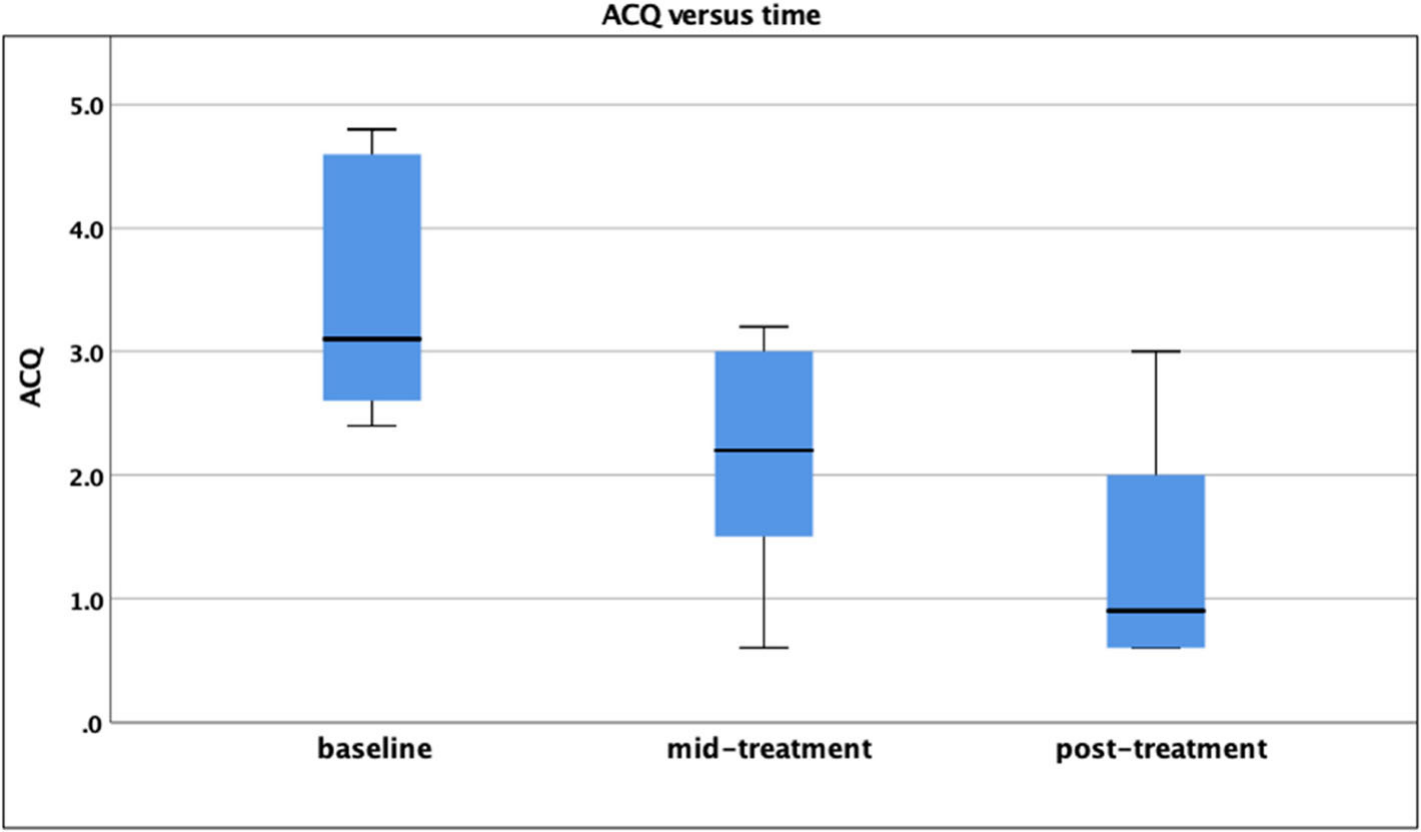
The progressive effect of BT on ACQ-5

As radiofrequency treatment accumulated, a dose-response was evident in ACQ-5 (Fig. 1). Using ANOVA, the overall effect was significant, Wilks’ lambda *p* = 0.004, partial eta squared 0.747, indicating a strong effect size.

**Fig. 2:**
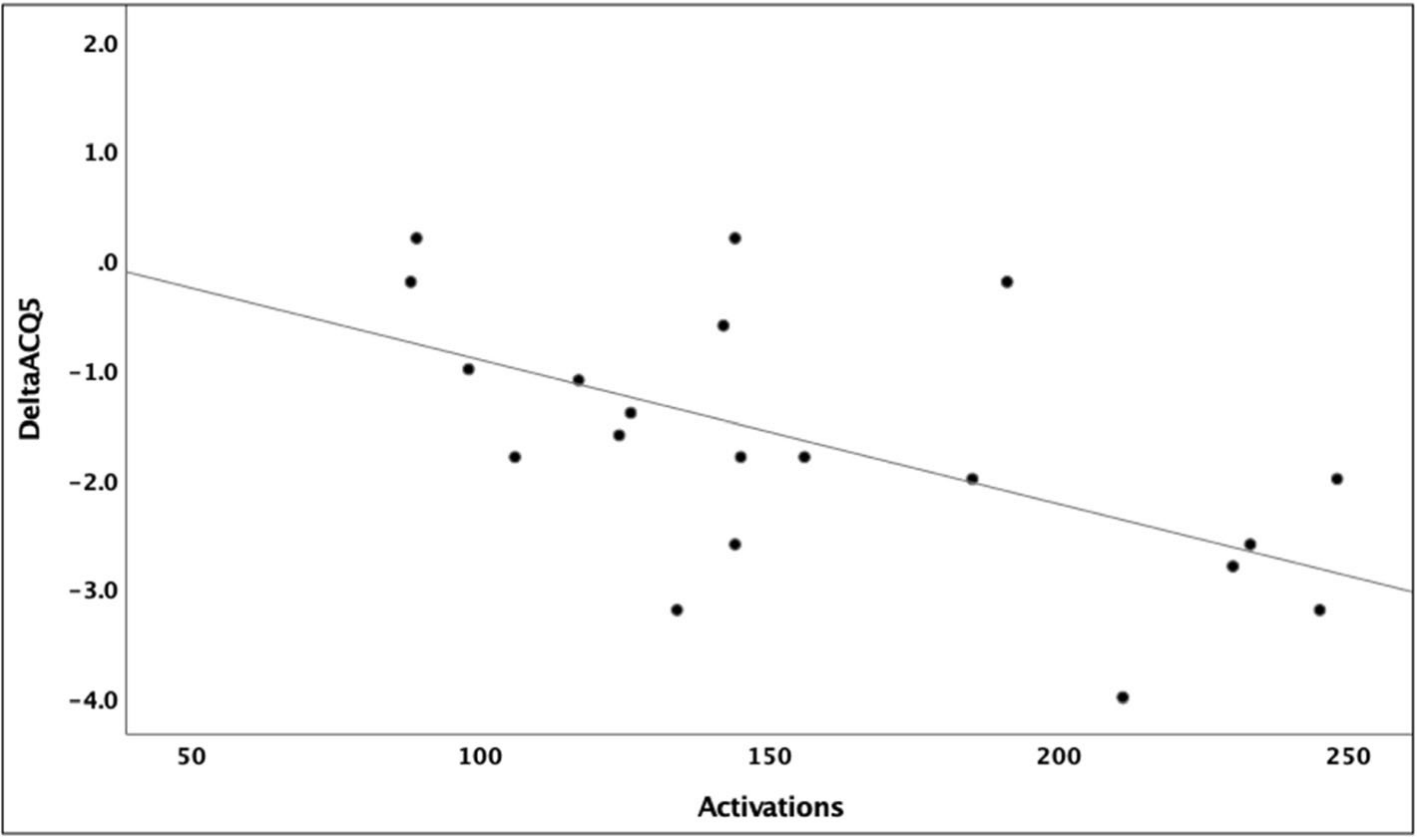
The relationship between RF activations and change in ACQ-5

The potential relationship between the radiofrequency treatment administered, and the response in the ACQ-5, was examined in the scatterplot, Fig. 2. The Pearson correlation was − 0.583, *p* < 0.01. The linear regression line was given by the equation y = 0.4–0.01x, where x = radiofrequency activations administered and y = change in ACQ-5 from baseline.

The effect of bronchial thermoplasty on lung function (spirometry and static lung volumes), measured at 3 time points, is shown in Table 1. Whilst no effect was observed in spirometry, treatment led to a significant reduction in the gas trapping measures, RV and RV/TLC.

**Table 1:** The effect of bronchial thermoplasty on lung function

	Baseline	Mid treatment	6w post	p
FEV1%predicted	42.9 ± 11.5	43.3 ± 12.3	46.0 ± 11.9	NS
VC % predicted	72.1 ± 12.8	74.5 ± 10.9	79.9 ± 13.4	NS
TLC % predicted	108.3 ± 20.7	109.9 ± 22.0	108.5 ± 22.0	NS
RV % predicted	158.8 ± 44.8	160.4 ± 51.4	148.0 ± 48	< 0.05
RV/TLC ratio %	56 ± 10	56 ± 11	52 ± 11	< 0.05

p:Anova repeated measures

### Imaging

At baseline, there were minor CT abnormalities detected in 8 of 10 patients. Bronchial wall thickening was reported in 4 cases, mild bronchiectasis in 2 cases and mild emphysema in 3 cases. The mean CT emphysema score, measured as percentage of Hounsfield units with a value less than minus 950 on the inspiratory scan at TLC, was 3.3 ± 5.0%. There was no evidence of any new CT abnormalities present on the mid-treatment CT scan.

The validity of the volume measurements made during the imaging studies was tested by comparing the CT estimated lung volumes at TLC and FRC, with the volumes measured by plethysmography. The Pearson correlation at TLC was r = 0.98, *p* < 0.001 and the offset difference between the two techniques (or c in the linear regression equation y = mx + c) was − 0.59 l - this difference being explained by the CT studies being performed in the supine position whilst plethysmography was conducted upright. At FRC, the correlation was r = 0.95, *p* < 0.001.

### Lobar volumes

The volumes of the individual lobes of each lung were calculated both at TLC and FRC, and then were compared before and after BT treatment of the left lung (right lung untreated control). The individual lobar volumes are shown in Table 2. A small reduction in lobar volume was evident after treatment of the left upper lobe, but no effect was observed otherwise in either lung.

**Table 2:** The effect of bronchial thermoplasty on lobar volumes

Lobe (litres)	TLC baseline	TLC Post left Rx	p	FRC baseline	FRC Post left Rx	p
Right Upper	1.04 ± 0.35	1.04 ± 0.35	ns	0.78 ± 0.25	0.78 ± 0.25	ns
Right Middle	0.37 ± 0.15	0.37 ± 0.15	ns	0.30 ± 0.10	0.30 ± 0.11	ns
Right Lower	1.26 ± 0.40	1.27 ± 0.40	ns	0.91 ± 0.26	0.94 ± 0.27	ns
Left Upper	1.24 ± 0.49	1.19 ± 0.45	< 0.05	0.90 ± 0.36	0.85 ± 0.36	0.12
Left Lower	1.03 ± 1.40	1.07 ± 2.02	ns	0.77 ± 0.29	0.77 ± 0.34	ns

### Airway luminal volumes

The low CT density of air was used to identify and reconstruct airways within the lung. Following automated segmentation of the bronchial tree, the airways were manually checked and corrected. The trachea and main bronchi were labelled central airways, being the airways untreated by BT. The lobar and segmental airways, and branches down to 2 mm in diameter were labelled distal airways, being the BT treated areas. In airways less than 2 mm in diameter, the distinction between intraluminal air and intra-alveolar air becomes difficult to resolve, and hence they are not included in the analysis. The volume of air in the above compartments was determined, and compared before and after BT treatment of the left lung. The validity of the airway volume measurements was tested by comparing the results with a database of age, sex and height matched control values for healthy adults held by FLUIDDA. When compared to the healthy airways, at baseline, asthmatic airways were smaller in volume but similar in order of magnitude: Total Airway Luminal Volume 71.4 ± 11.9% predicted (*p* < 0.05).

A significant increase in volume was observed after treatment in the airways of the left lung at both TLC and at FRC (Table 3). The magnitude of the increase in airway volume at TLC was 27%, and at FRC was 17%. Increased luminal volume was observed on the treated side in 9 of 10 patients at both TLC and FRC. No change in airway volume was seen in the untreated right side, nor the central airways.

**Table 3:** The effect of BT on airway luminal volumes

Airway volume (mls)	TLC baseline	TLC Post left Rx	p	FRC baseline	FRC Post left Rx	p
Distal Left side	4.8 ± 2.1	6.1 ± 3.3	< 0.05	2.3 ± 1.0	2.7 ± 1.2	< 0.01
Distal Right side	5.6 ± 3.3	5.8 ± 4.4	ns	2.8 ± 1.5	2.7 ± 1.6	ns
Central airways	24.5 ± 8.6	24.6 ± 9.9	ns	16.5 ± 6.3	17.5 ± 6.4	ns

p:paired t-test

The ratios between the distal airway volume and the lung volume at FRC, pre and post treatment are shown in Table 4. The distal airway volume as a proportion of the lung volume is shown to increase after treatment.

**Table 4:** Ratio of distal airway volume (mls) to lung volume (litres) at FRC

	Baseline	Post Left Rx	p
Left Lung	1.48 ± 0.72	1.82 ± 1.04	< 0.05
Right Lung	1.09 ± 0.43	1.08 ± 0.55	NS

p:paired t-test

**Fig. 3:**
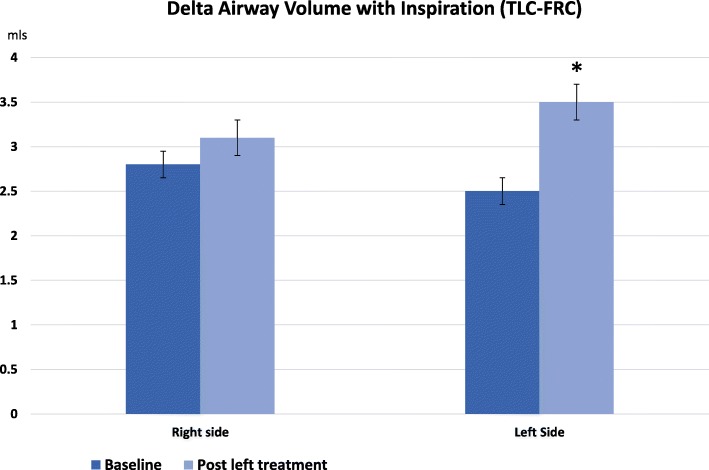
Change in airway distensibility after BT

The change in airway volume with full inspiration from FRC was compared in both lungs before and after treatment (Table 5). The data suggests an increase in airway distensibility after treatment, not seen on the control side (Fig. 3).

**Table 5:** Inspiratory change in airway volume pre and post treatment

Delta Airway Volume TLC minus FRC (mls)	Baseline	Post Left Rx	Mean change from baseline
Left lung	2.5 ± 1.3	3.5 ± 2.5	48 ± 79%^*^
Right Lung	2.8 ± 2.3	3.1 ± 3.1	5 ± 41%

* = *p* < 0.05, paired t-test, left side versus right side

The potential relationship between the change in luminal airway volume at TLC on the treated left side, and the change in ACQ-5 at the mid treatment evaluation was explored. The Pearson Correlation was r = −0.61, *p* = 0.06.

### Airway resistance

Airway resistance was estimated using computational fluid dynamics (11,12,16) and was reported for each lobe at TLC and FRC, and pre and post treatment of the left side. We were unable to demonstrate a statistically significant effect.

### Additional measurements

Air trapping was defined as the percentage intrapulmonary voxels ranging between − 1024 and − 850 Hounsfield units measured in expiration at FRC [20]. No effect was observed following BT. No significant effects of treatment were observed on airway wall volume, nor airway wall area. Blood vessel density was determined by using a density mask of − 600 to + 600 Hounsfield units applied to the TLC scan. The total estimated blood vessel volume at baseline was 77.5 ± 46.5 mls for the right lung and 68.8 ± 39.7 for the left lung. No changes were observed after in either value after BT.

## Discussion

The most important finding in this study is the demonstration that airway luminal volume substantially increased after BT. This observation provides the pathophysiological missing link between the reduction in airway smooth muscle mass observed in biopsy studies, and the improvement in symptoms observed after treatment. It is a novel observation in humans but is entirely consistent with early canine preclinical studies [21] where one lung was treated by BT, whilst the other served as a control. Following recovery, the dogs were anaesthetised, ventilated and paralysed, and then the lungs were inflated to pre-specified inflation pressures. At each pressure a CT scan was performed, and the airway luminal area determined. The authors demonstrated that for any individual inflation pressure, the airways of the treated lung were significantly larger than on the untreated side [21].

In addition to showing that after BT, airway luminal volume is greater than control at the same level of lung inflation, the current study suggests that the airways may be more distensible after BT. The incremental increase in airway volume as the patient inspires from FRC to TLC is small on the untreated side, but much greater in the treated lung. Reduced airway distensibility is a known feature of the asthmatic airway, ascribed to airway wall remodelling [22, 23]. BT has been shown to reduce the hypertrophied airway smooth muscle layer –a characteristic feature of airway remodelling in severe asthma [24, 25].

It would be anticipated that increased luminal volume would have downstream effects in more distal parts of the lung. This has been modelled by Donovan [26], who has predicted a reopening cascade in distal airways following BT. It would therefore be expected that a deflating effect would be observed in lungs that exhibit marked gas trapping, and, indeed, that was seen in this current study, where both the RV and RV/TLC ratio reduced after treatment. We also have noted this observation previously in a larger series [27]. This effect is also consistent with emerging data from MRI studies showing improvement in ventilation heterogeneity in severe asthma following BT [28]. With the increased luminal airway volume, it would also be expected that a reduction in airway resistance would be observed. We were unable to show this in this study but we believe that this is likely to be due to the small sample size and the large biological variation between patients in this heavily calculated parameter.

The current study also demonstrates, for the first time, a progressive improvement in symptoms as treatment accumulates. This is an important observation because the demonstration of a dose –response relationship to a therapy is one of the Bradford Hill criteria used to assess biological causality, and argues in favour of BT having a physiological effect rather than a placebo effect [29]. The relationship demonstrated between radiofrequency activations delivered, and improvement in symptoms observed, further supports previous work suggesting that a potential reason for non-response to BT is an inadequate quantum of radiofrequency treatment [30].

Despite this being a small study, a trend emerged suggesting that the improvement in symptoms at the mid treatment evaluation was related to the increase in airway volume observed on the treated side. This fits with clinical expectations but will need a larger study to prove. Of the 10 patients in the current study, 9 showed an improvement in ACQ-5 greater than the minimal clinically significant difference at the mid treatment evaluation. The remaining tenth patient, however, showed improvement in FEV1% predicted, Residual Volume and left–sided airway volumes. The treating physician therefore concluded that the patient had responded to therapy, although this had not been reflected in the ACQ-5 score. This demonstrates the perceptual element to the ACQ-5 score. The ability to quantify a change in airway volume with treatment, now provides a potential objective marker of response to therapy which could be a useful tool in future clinical practice.

## Conclusion

In this human study, we demonstrate that bronchial thermoplasty increases the luminal airway volume on the treated side compared to the control lung. We suggest that this is the link between the airway smooth muscle atrophy demonstrated pathologically, and the improvement in symptoms observed clinically.

## Data Availability

The data sets analysed in this study are available from the corresponding author upon request.

## Abbreviations

ACQ-5: Asthma control questionnaire-5 item version
BT: Bronchial thermoplasty
CT: Computerized tomography
ERS/ATS: European Respiratory Society/American Thoracic Society
FEV1: Forced expiratory volume in 1 s
FRC: Functional Residual Capacity
RV: Residual Volume
TLC: Total Lung Capacity
VC: Vital capacity
